# Inhibitory Effect of Lidocaine on Duodenal Peristalsis During Endoscopic Retrograde Cholangiopancreatography: A Multicenter, Randomized Controlled Trial (With Video)

**DOI:** 10.1002/deo2.70252

**Published:** 2025-11-27

**Authors:** Masato Suzuki, Yusuke Sekino, Kunihiro Hosono, Yusuke Kurita, Hiroki Uechi, Kento Kuzuu, Shiori Uchiyama, Kenichi Kawana, Hajime Nagase, Kensuke Kubota, Masato Yoneda, Atsushi Nakajima

**Affiliations:** ^1^ Department of Gastroenterology National Hospital Organization Yokohama Medical Center Kanagawa Japan; ^2^ Department of Gastroenterology Yokohama Rosai Hospital Kanagawa Japan; ^3^ Department of Gastroenterology Yokosuka General Medical Center Kanagawa Japan; ^4^ Department of Gastroenterology and Hepatology Yokohama City University Graduate School of Medicine Kanagawa Japan; ^5^ Department of Hepatobiliary and Pancreatic Medicine NTT Medical Center Tokyo Tokyo Japan; ^6^ Center For Digestive Disease and Inflammatory Bowel Disease (IBD) Ofuna Chuo Hospital Kanagawa Japan; ^7^ Department of Gastroenterology Sagamihara Municipal Hospital Kanagawa Japan

**Keywords:** elderly, endoscopic retrograde cholangiopancreatography, lidocaine, peristalsis, randomized controlled trial

## Abstract

**Objectives:**

Conventional antispasmodics used during endoscopic retrograde cholangiopancreatography (ERCP), such as hyoscine butylbromide and glucagon, are often contraindicated in elderly patients with comorbidities. This trial aimed to assess the efficacy of lidocaine for inhibiting duodenal peristalsis during ERCP.

**Methods:**

This multicenter randomized controlled trial enrolled 40 elderly patients (aged 65–89 years) who were scheduled to undergo ERCP. Patients were randomly assigned to the lidocaine group or the control group using a computer‐generated sequence with stratification by age and sex. The lidocaine group (*n* = 19) received 2% lidocaine jelly mixed with saline, while the control group (*n* = 21) received a placebo jelly mixed with saline. The primary endpoint was inhibition of duodenal peristalsis. Secondary endpoints included the required time from drug spraying to cessation of duodenal peristalsis, stop duration time (DT) from cessation of peristalsis until peristalsis recovery, and adverse events.

**Results:**

Eighteen patients from the lidocaine group and 19 patients from the control group were analyzed for the primary outcome. The inhibition rate of duodenal peristalsis was significantly higher in the lidocaine group (94.4%) than in the control group (52.6%) (*p* = 0.008). The required time was significantly shorter in the lidocaine group than in the control group (*p* < 0.001). No significant difference was observed in the stop DT (*p* = 0.862); no adverse events occurred in either group.

**Conclusions:**

In our limited cohort, lidocaine inhibited duodenal peristalsis during ERCP without adverse events, suggesting its potential as a safe and practical option (jRCT No. 031190059).

## 1 Introduction

Endoscopic retrograde cholangiopancreatography (ERCP) is a widely performed procedure used for biliary and pancreatic diseases [1, 2]. Although duodenal peristalsis plays an important physiological role, excessive or strong peristalsis is often observed to interfere with the establishment of a stable visual field during ERCP. Conventionally, antispasmodic agents such as hyoscine butylbromide and glucagon are used to overcome this challenge [3]. However, these agents are associated with various adverse events [4, 5]. Furthermore, hyoscine butylbromide is contraindicated in patients with severe cardiac disease, and glucagon should be used with caution in individuals with diabetes mellitus [6]. Therefore, these drugs can be difficult to use, particularly in elderly patients who often have such comorbidities.

Lidocaine, a local anesthetic, has been shown to inhibit colonic peristalsis by acting on mucosal nerves [7]. However, its effect on duodenal peristalsis during ERCP remains unclear.

In Japan, lidocaine‐based jelly preparations are commonly used for topical anesthesia. If lidocaine can be shown to effectively inhibit duodenal peristalsis, it could offer a safe and practical option, particularly in patients for whom conventional antispasmodics are contraindicated.

To investigate this possibility, we conducted a randomized controlled trial to evaluate the inhibitory effect of lidocaine jelly on duodenal peristalsis. In this trial, we compared lidocaine jelly with a placebo jelly composed of a water‐based gel containing citric acid.

## 2 Methods

### 2.1 Trial Design and Setting

This was a multicenter, randomized, placebo‐controlled trial with a parallel‐group design and a 1:1 allocation ratio. The trial was conducted at three hospitals in Japan:

### 2.2 Ethical Considerations

The trial protocol complied with the Declaration of Helsinki and Japanese ethical guidelines for clinical research. It was classified as a Specified Clinical Trial under the Clinical Trials Act of Japan and approved by the Certified Review Board of Yokohama City University Hospital on July 5, 2020 (CRB 3180007). The trial was registered in the Japan Registry of Clinical Trials (jRCT No.031190059). Written informed consent was obtained from all participants, and the Consolidated Standards of Reporting Trials guidelines were followed.

### 2.3 Eligibility Criteria

Patients scheduled to undergo ERCP at one of the three participating hospitals were eligible for enrollment. Patients were included in the study according to the following criteria: (1) age between 65–89 years, (2) scheduled for ERCP, (3) duodenal peristalsis score of 1–3, and (4) provided written informed consent. The exclusion criteria were as follows: (1) acute pancreatitis, (2) known allergy to amide‐type local anesthetics, (3) altered or postsurgical upper gastrointestinal anatomy, (4) pregnancy, possibility of pregnancy, or lactation, (5) cardiac conduction disorders, (6) severe hepatic or renal dysfunction, (7) duodenal peristalsis score of 4 or 5, and (8) judged ineligible by the investigators.

### 2.4 Intervention and Randomization

Duodenal peristalsis was continuously evaluated throughout ERCP after the papilla was brought into en face view. When the peristalsis score reached 1–3, eligible patients were randomly assigned to either the lidocaine group (L group) or the control group (C group). Investigators submitted patient information to the coordinating center via facsimile or email. After verifying eligibility, the coordinating center performed randomization using a computer‐generated sequence with stratification by age and sex. Allocation was concealed from the patients and independent assessors but not from the drug administrators or endoscopists.

### 2.5 ERCP Procedure

Pharyngeal anesthesia was achieved by administering five sprays of 8% lidocaine spray (Sandoz K.K., Tokyo, Japan). The ERCP procedure was then performed by one of three experienced endoscopists (Masato Suzuki [MS], Hiroki Uechi [UH], and Kento Kuzuu [KK]), each with experience of performing more than 200 ERCP procedures. All participants received intravenous midazolam and pentazocine before the procedure. Heart rate, blood pressure, electrocardiography, and peripheral oxygen saturation were monitored throughout the procedure. Endoscopists used a lubricant gel without lidocaine on the scope, and carbon dioxide insufflation was used for all patients. All ERCP procedures were recorded on video.

### 2.6 Trial Endpoints

The primary endpoint was the inhibitory effect of lidocaine on duodenal peristalsis during ERCP, which was measured using a scoring system. The scoring system was divided into five grades based on the strength of the peristalsis and the difficulty of ERCP procedures [8]: (1) duodenal peristalsis is too strong to perform ERCP procedures; (2) strong duodenal peristalsis making ERCP procedures difficult; (3) moderate duodenal peristalsis, but ERCP procedures can be performed; (4) easy to perform ERCP procedures due to slight duodenal peristalsis; and (5) easy to perform ERCP procedures due to no duodenal peristalsis. Duodenal peristalsis was continuously scored throughout the ERCP procedure, starting from when an en face view of the papilla was obtained. After spraying the trial drug, scores of 4 or 5 were considered indicative of a peristaltic inhibitory effect, whereas scores of 1–3 were considered to indicate no inhibitory effect.

Patients in the L group received 10 mL of lidocaine jelly (2%) mixed with 10 mL of saline, while those in the C group received 10 mL of a citric acid‐based placebo gel mixed with 10 mL of saline. Each solution was warmed to 36°C and sprayed onto a 5‐cm segment of the duodenal mucosa surrounding the major papilla using the endoscope's accessory channel. The endoscopists observed the degree of duodenal peristalsis for up to 3 min after the drug was sprayed. The ERCP procedure continued without interruption during this observation period. If a peristaltic inhibitory effect was observed after 3 min, it was not considered to be due to the drug.

**FIGURE 1:**
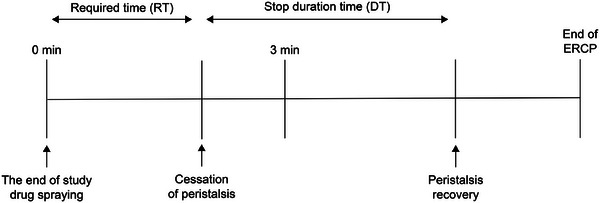
Timetable of observation during the ERCP procedure. ERCP, endoscopic retrograde cholangiopancreatography.

The secondary endpoints were (1) required time (RT) from drug spraying to cessation of duodenal peristalsis; (2) stop duration time (DT) from cessation of peristalsis until peristalsis recovery [9] (Figure 1); and (3) adverse events (AEs), monitored by the investigator on days 1, 2, 3, and 28 (with a permissible widow of days 21–35).

Duodenal peristalsis was evaluated continuously throughout ERCP using a predefined 5‐point scoring system. RT and DT were measured based on changes in this score: peristalsis recovery was defined as the first time point when the score returned from 4 or 5 (inhibition) to 1–3 (no inhibition). AEs were graded according to the Common Terminology Criteria for Adverse Events v5.0.

Furthermore, step‐specific procedure times (cannulation, papillary intervention, stent placement, and biliary stone removal) were explored, with both step duration and spray‐to‐completion time being recorded. Analyses were performed only when ≥10 patients in total and ≥5 per group were available, as an exploratory analysis to obtain more objective procedural insights. ERCP‐related AEs were also documented descriptively and defined according to Cotton et al. [10].

After all cases were enrolled, three independent blinded expert endoscopists (independent assessors: KK, YS, and SU) reviewed the video recordings. For the primary endpoint, each assessor independently evaluated peristalsis; in case of disagreement, a majority vote determined the result. For the RT and DT, the mean of the three evaluations was used. Although time‐based exclusion criteria were initially defined to ensure procedural quality, these were removed to avoid potential bias.

### 2.7 Drug Supply

Lidocaine jelly (Xylocaine Jelly 2%) was obtained from Sandoz K.K. (Tokyo, Japan), and the placebo, PG Water EJ, was obtained from Terumo Corporation (Tokyo, Japan). The placebo consisted of a water‐based gel containing citric acid. After randomization, the trial drug administrators (YO and HN) mixed 10 mL of the assigned trial agent with 10 mL of saline.

### 2.8 Sample Size Calculation

Nemoto et al. [11] reported a 96.7% spasm inhibition rate with 30 mL of 2% lidocaine solution in the colon, and Fujinami et al. [9] reported a 0% spasm inhibition rate with 50 mL of warm water in the duodenum [9]. Based on these reports and the current trial, we estimated that the spasm inhibition rate in the duodenum would be 70% with lidocaine and 10% with placebo. To detect the inhibitory effect of lidocaine using Fisher's exact test with a two‐sided alpha error of 0.05 and a power of 0.90, 16 patients per group were required. Assuming a dropout rate, we aimed to enroll 20 patients per group.

### 2.9 Statistical Methods

Fisher's exact test was used to compare categorical variables between two groups. The Mann‐Whitney *U* test was used to compare continuous variables. The median RT and DT were calculated, and Kaplan–Meier analysis was performed with log‐rank testing to compare time‐related outcomes. All analyses were performed using EZR software (64‐bit version; Jichi Medical University, Saitama, Japan), with statistical significance set at *p* < 0.05.

### 2.10 Data Monitoring

A single monitoring supervisor was appointed at the Department of Gastroenterology and Hepatology, Yokohama City University Hospital, the lead institution of this trial. The supervisor conducted central monitoring throughout the trial, overseeing subject enrollment status, eligibility of enrolled participants, evaluation of AEs, and identification of any major deviations from the trial protocol or procedures.

## 3 Results

### 3.1 Patients

**FIGURE 2:**
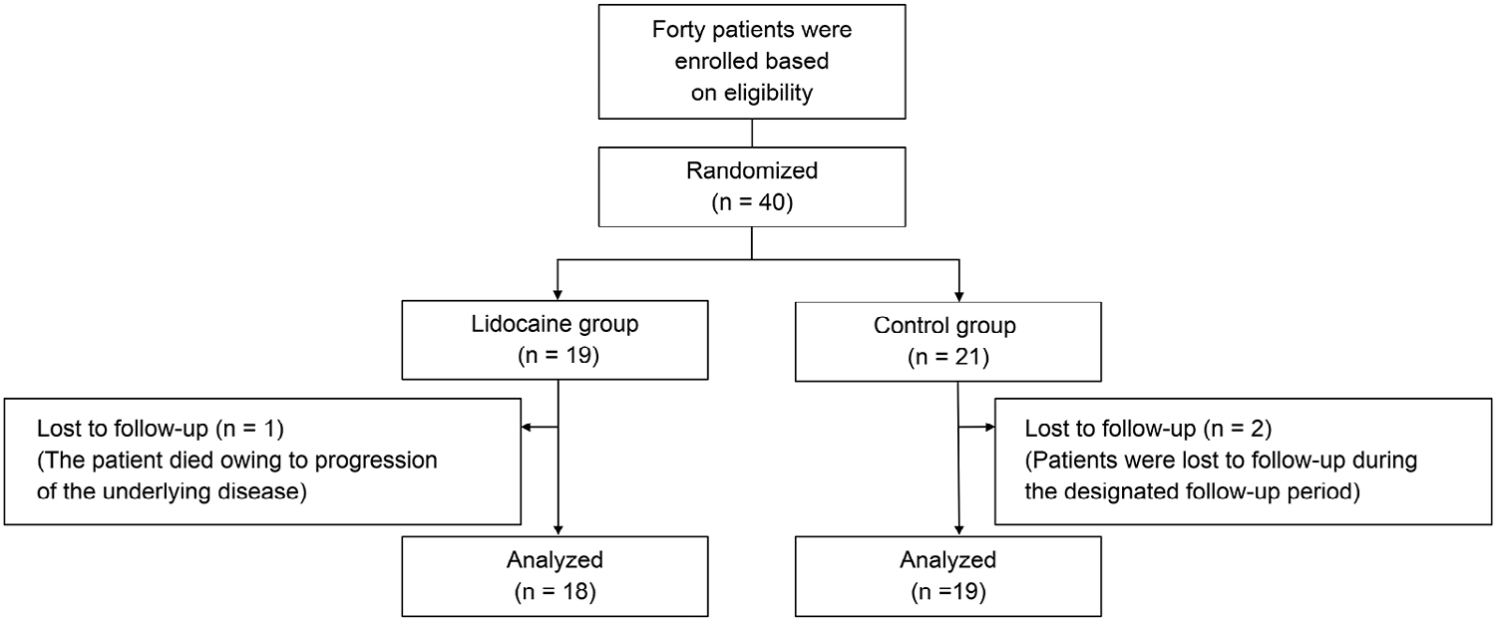
Trial flow chart. ERCP, endoscopic retrograde cholangiopancreatography.

Participants were recruited and randomized between October 7, 2019, and September 27, 2024. Follow‐up for the last enrolled participant was completed on October 17, 2024 (Figure 2). These patients underwent ERCP and were allocated to either the L group (*n* = 19) or the C group (*n* = 21).

In the L group, one patient was lost to follow‐up owing to disease progression. In the C group, two patients were lost to follow‐up after failing to visit during the specified observation period. Consequently, 18 patients from the L group and 19 patients from the C group were included in the final analysis.

Table 1 presents the baseline characteristics and details of the ERCP procedures. Except for the peristalsis score after drug administration, no other variables demonstrated significant intergroup differences. The peristalsis score was significantly higher in the L group than in the C group (p = 0.008).

**TABLE 1:** Baseline patient characteristics and endoscopic retrograde cholangiopancreatography (ERCP) procedure details.

	Lidocaine group (*n* = 18)	Control group (*n* = 19)	*p*‐Value
**Patient characteristics**			
Age (years)	73 (70.3–80.0)	73 (70.0–78.5)	0.715
Sex (female)	8 (44.4)	9 (47.4)	1.000
Body mass index (kg/m^2^)	23.2 (20.3–27.8)	23.8 (21.4–26.1)	1.000
**Blood test results**			
White blood cells (×1,000/µL)	6.4 (5.3–8.3)	5.7 (5.2–6.9)	0.484
C‐reactive protein levels (mg/dL)	0.8 (0.1–2.5)	1.0 (0.2–3.4)	0.447
Total bilirubin (mg/dL)	0.9 (0.5–1.1)	0.9 (0.6–1.9)	0.345
AST (U/L)	20.0 (16.3–64.8)	28.0 (21.5–52.0)	0.287
ALT (U/L)	30.5 (10.3–68.8)	56.0 (18.5–91.5)	0.331
AMY (U/L)	75.5 (62.0–101.0)	63.0 (44.0–112.0)	0.421
**ERCP details**			
Naïve papilla	10 (55.6)	13 (68.4)	0.508
Parapapillary diverticulum	6 (33.3)	5 (26.3)	0.728
Reason for ERCP			
Biliary stone removal	12 (66.7)	13 (68.4)	1.000
SPACE	2 (11.1)	3 (15.8)	1.000
Biliary drainage	3 (16.7)	2 (10.5)	0.660
Other	1 (5.6)	1 (5.3)	1.000
Cannulation success	18 (100)	19 (100)	1.000
Procedure time (minutes)	26.0 (18.3–29.8)	21.0 (14.0–31.5)	0.447
Total ERCP‐related adverse events	1 (5.6)	1 (5.3)	1.000
Post‐ERCP pancreatitis	1 (5.6)	1 (5.3)	1.000
**Indication for study drug use**			
Canulation	2 (11.1)	2 (10.5)	1.000
Papillary intervention	6 (33.3)	7 (36.8)	1.000
Stent placement	3 (16.7)	4 (21.1)	1.000
Biliary stone removal	7 (38.9)	6 (31.6)	0.737
Peristalsis score before dispersing the study drug	2.0 (2.0–2.0)	2.0 (2.0–2.0)	1.000
Peristalsis score after dispersing the study drug	5.0 (5.0‐5.0)	3.0 (2.5‐5.0)	0.008

Data are presented as the median (IQR) or *n* (%), unless otherwise specified.

Abbreviations: ALT, alanine aminotransferase; AMY, amylase; AST, aspartate aminotransferase; ERCP, endoscopic retrograde cholangiopancreatography; IQR, interquartile range; *n*, number of participants; SPACE, serial pancreatic juice aspiration cytological examination.

### 3.2 Primary Endpoints

Table 2 summarizes the inhibitory effect of lidocaine and the placebo on duodenal peristalsis. Notably, the inhibitory effect was observed in 94.4% (17/18) of patients in the L group and 52.6% (10/19) in the C group, demonstrating that the L group had a significantly higher rate of duodenal peristalsis inhibition than the C group (*p* = 0.008).

**TABLE 2:** Comparison of the inhibitory effect on duodenal peristalsis between the treatment and control groups.

	Lidocaine group (*n* = 18)	Control group (*n* = 19)	*p*‐Value
Inhibitory effect on duodenal peristalsis, *n* (%)	17 (94.4)	10 (52.6)	0.008

Abbreviation: *n*, number of participants.

Video S1 shows representative video footage from a patient in the L group. After the solution containing 10 mL of lidocaine jelly was sprayed, duodenal peristalsis ceased immediately.

### 3.3 Secondary Endpoints

**FIGURE 3:**
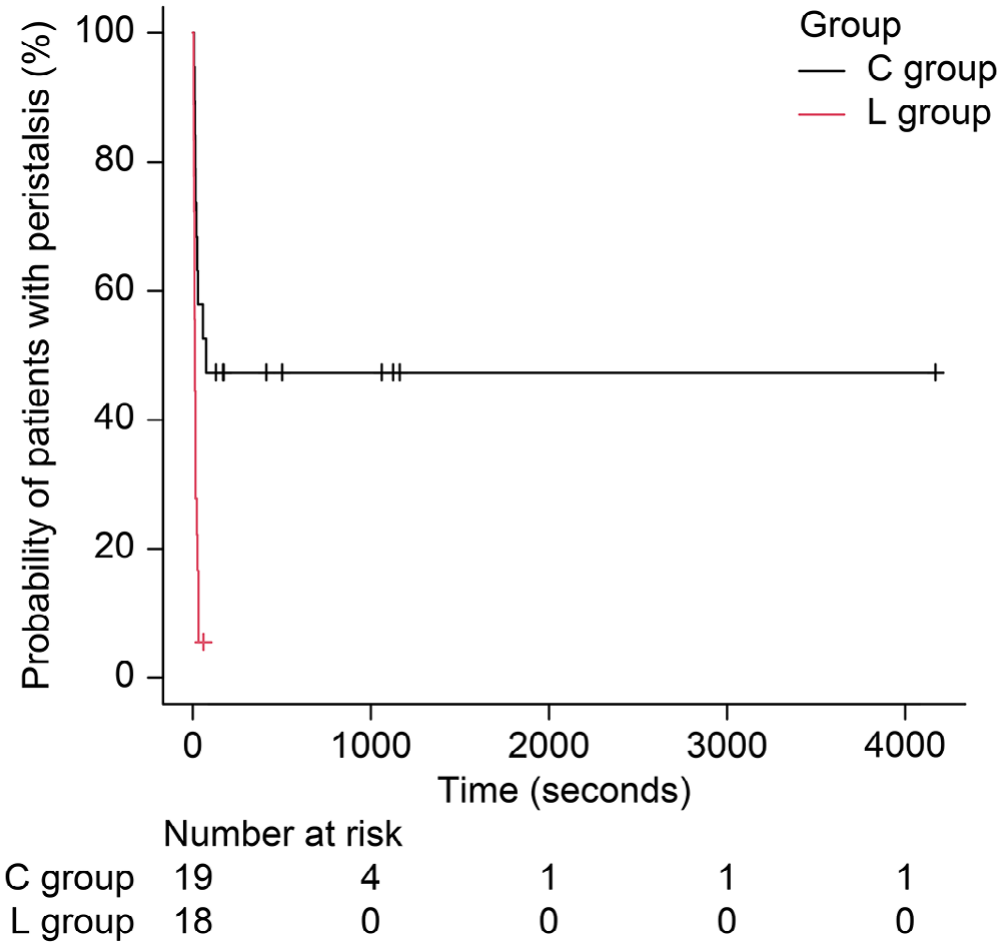
Comparison of the required time (RT) for inhibited peristalsis between the L and C groups. C group, control group; L group, lidocaine group.

Figure 3 shows Kaplan–Meier estimates for the RT in both L and C groups. The median RT was 12 s (95% confidence interval [CI], 8–15 s) in the L group and 75 s (95% CI, 15 s–not applicable [NA]) in the C group, with a significant difference between the two groups (*p* = 0.002). “NA” was used when the upper limit of the CI could not be estimated due to censoring.

**FIGURE 4:**
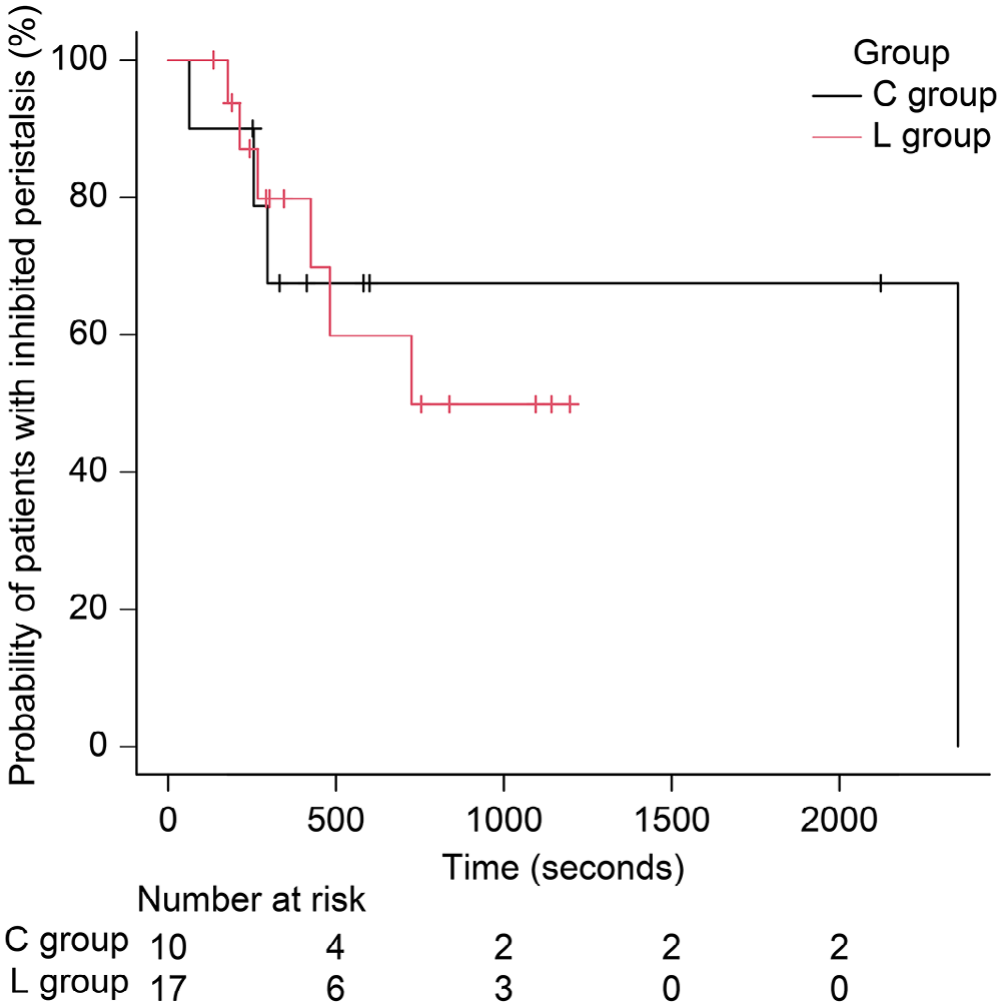
Comparison of the stop duration time (DT) for inhibited peristalsis between the L and C groups. C group, control group; L group, lidocaine group.

Figure 4 shows Kaplan–Meier estimates for the DT in both L and C groups. The median DT was 725 s (95% CI, 267–NA) in the L group and 2,353 s (95% CI, 63–NA) in the C group, with no statistically significant difference between the two groups (*p* = 0.862).

No AEs were reported in any participants throughout the trial period.

### 3.4 Exploratory Analysis

Step‐specific procedure times are shown in Tables 3 and 4. For papillary intervention, both measures tended to be shorter with lidocaine but did not reach statistical significance. For biliary stone removal, times were comparable between the groups.

**TABLE 3:** Comparison of step‐specific procedure times between the treatment and control groups for papillary intervention.

	Lidocaine group (*n* = 6)	Control group (*n* = 7)	*p*‐value
**Papillary intervention (seconds)**			
Step duration time	73.5 (63.0–119.3)	158.0 (118.0–171.5)	0.138
Spray‐to‐completion time	133.5 (120.5–165.3)	186.0 (169.0–247.0)	0.295

Data are presented as the median (IQR).

Abbreviation: *n*, number of participants.

**TABLE 4:** Comparison of step‐specific procedure times between the treatment and control groups for biliary stone removal.

	Lidocaine group (*n* = 7)	Control group (*n* = 6)	*p*‐value
**Biliary stone removal (seconds)**			
Step duration time	292.0 (192.5–452.5)	354.0 (156.3–547.3)	1.000
Spray‐to‐completion time	307.0 (183.0–425.0)	315.5 (140.8–573.5)	1.000

Data are presented as the median (IQR).

*n*, number of participants.

## 4 Discussion

To the best of our knowledge, this randomized trial is the first to evaluate the effect of lidocaine on duodenal peristalsis during ERCP, using a non‐pharmacologic hydrating jelly as the control. Our findings demonstrated that lidocaine significantly inhibited duodenal peristalsis, with an inhibition rate of 94.4% when using lidocaine and 52.6% when using the placebo. These results suggest that lidocaine may have a potential role as a safe and effective agent during ERCP.

Conventionally, antispasmodic agents such as hyoscine butylbromide and glucagon have been used to inhibit duodenal peristalsis during ERCP [3]. Hyoscine butylbromide blocks muscarinic M2 and M3 receptors—located at the smooth muscle and epithelial cells—which induce smooth muscle contraction and secretion, respectively [12]. However, its use is associated with a range of AEs, including glaucoma, tachycardia, and hypotension [5]. Although glucagon is also effective [13, 14], it can cause electrolyte imbalances such as hyperkalemia and hyperglycemia [15].

These limitations are particularly relevant in elderly patients, who are the primary candidates for ERCP. This population often has cardiovascular complications [16] and is more likely to have diabetes due to impaired insulin secretion or insulin resistance [17, 18]. Therefore, safer pharmacologic treatments are especially desirable in this high‐risk group.

In the present study, no AEs occurred with duodenal lidocaine spraying, suggesting a favorable safety profile. From a cost perspective, hyoscine butylbromide is priced similarly to lidocaine, whereas glucagon is substantially more expensive [19]. Thus, lidocaine may represent a practical, cost‐conscious option, particularly compared with glucagon.

The effectiveness of peppermint oil [20] and Shakuyakukanzoto [9, 21] for inhibiting peristalsis during ERCP has also been reported, and both show inhibition without serious AEs [9, 20, 21]. However, available data remain limited, and further studies are warranted to confirm their clinical consistency. Moreover, these agents are not consistently available in all endoscopy units—Shakuyakukanzoto is typically not stocked, and access to peppermint oil varies between institutions. In contrast, lidocaine is routinely stocked in many endoscopy units due to its common use for topical anesthesia in various endoscopic procedures. This makes it readily accessible and immediately applicable when duodenal peristalsis occurs during ERCP. Although the use of lidocaine jelly in this context is off‐label, its practicality and availability may offer distinct advantages over peppermint oil and Shakuyakukanzoto in routine clinical settings.

Previous studies have demonstrated that lidocaine can inhibit peristalsis in the colon during colonoscopy [7, 11]. However, its impact on duodenal peristalsis and its safety profile in the context of ERCP had not been clearly established prior to this trial. Notably, neither this trial nor the previous reports [7, 11] have documented any AEs associated with the spraying of lidocaine. These findings suggest that spraying lidocaine into the gastrointestinal tract may be safe for all patients, not just the elderly.

The RT was significantly shorter in the lidocaine group, whereas DT did not differ significantly between the groups. The median DT in our trial was 725 s, which is considerably longer than the 227 s reported by Nemoto et al. [11]. This may also stem from differing definitions, as prior studies defined “rebound spasm” as resumption within 5 min, while our trial considered it as any resumption during the ERCP procedure. Only single‐dosing was tested; given the absence of AEs, cautious re‐spraying may be acceptable but remains unproven. Regarding AEs as a secondary endpoint, none were observed in either group. These results support the safety and feasibility of the use of lidocaine as an adjunct for ERCP, especially in elderly patients with comorbidities.

In the papillary intervention subgroup, time‐based outcomes favored lidocaine but were not statistically significant, likely owing to the small sample size.

Despite its strengths, this trial had several notable limitations. First, the spraying of lidocaine jelly onto the duodenal mucosa represents an off‐label use of lidocaine. Lidocaine jelly is approved for topical surface anesthesia [22], and its intraduodenal use should be approached cautiously until further supportive data are available. Second, the jelly used as a placebo was a glucose‐containing hydrating jelly. We intentionally avoided using normal saline alone as the placebo, as its lack of gel‐like consistency appearance, would have made it easily distinguishable from the lidocaine jelly, thereby compromising blinding. This posed a potential risk of introducing observer bias, particularly because peristalsis inhibition was evaluated by independent assessors based on endoscopic videos. To maintain adequate blinding, we chose a glucose‐containing hydrating jelly with a similar appearance. Although it has no known pharmacologic effects, a relatively high rate of peristalsis inhibition (52.9%) was observed in the control group. High caloric contents are known to inhibit gastric motility, possibly via nutrient‐induced small intestinal feedback [23]. Therefore, the caloric content of the placebo jelly, derived from glucose, may have contributed to the suppression of duodenal peristalsis observed in this group, potentially limiting its suitability as a true inert placebo. Future studies should consider alternative placebo materials that maintain blinding while minimizing physiological effects on gastrointestinal motility. Third, the scoring of peristalsis inhibition was subjective and based on procedural difficulty. While we attempted to minimize bias through the use of independent assessors, individual operator skill could still influence evaluations. A more objective and reproducible assessment method would strengthen future studies. Fourth, this was an open‐label trial, introducing potential bias. A double‐blind, placebo‐controlled trial design would be ideal to validate these findings and eliminate residual sources of bias. Finally, the sample size was small (n = 37), which was sufficient to demonstrate proof of concept but insufficient to detect rare complications or long‐term safety outcomes, limiting generalizability.

In conclusion, lidocaine significantly inhibited duodenal peristalsis compared with placebo, without AEs in this limited cohort, suggesting its potential as a safe and practical agent for ERCP.

## Author Contributions

**Masato Suzuki** and **Yusuke Sekino** contributed to the conceptualization of the trial. **Masato Suzuki** and **Kunihiro Hosono** conducted the feasibility phase and were responsible for participant recruitment and follow‐up. **Masato Suzuki** performed the data analysis and interpretation. ERCP procedures were carried out by **Masato Suzuki**, **Hiroki Uechi (UH)**, and **Kento Kuzuu (KK)**. Independent assessments were conducted by **Kenichi Kawana**, **Yusuke Sekino**, and **Shiori Uchiyama**. **Yusuke Kurita**, **Hajime Nagase**, **Kensuke Kubota**, **Masato Yoneda**, and **Atsushi Nakajima** contributed to the study design, data interpretation, and critical revision of the manuscript.

## Funding

The authors have nothing to report.

## Ethics Statement

This trial was approved by the Certified Review Board of Yokohama City University Hospital on July 5, 2020 (approval number: CRB3180007).

## Conflicts of Interest

The authors declare no conflicts of interest.

## Consent

Informed consent was obtained from all participants.

## Clinical Trial Registration

This trial was registered with the Japan Registry of Clinical Trials (jRCT No. 031190059).

## Supporting information




**VIDEO S1**: Before spraying the lidocaine jelly and saline mixture, duodenal peristalsis was observed. After spraying the solution, duodenal peristalsis was markedly inhibited.
Supporting Information
